# Supplementary material for: Using passive sensor data to probe associations of social structure with changes in personality: A synthesis of network analysis and machine learning
Source: PLoS One. 2022 Nov 30;17(11):e0277516. doi: 10.1371/journal.pone.0277516 (PMC9710841; doi:10.1371/journal.pone.0277516)
Supplement: S2 File — (DOCX) [file pone.0277516.s002.docx]

S2_File

# NETWORK CONSTRUCTION AND FEATURE EXTRACTION SCRIPT[¶](#X8c0db938a68430e4c8e28ac68e4c4eaf5053481)

### Written solely for research contained within the manuscript:[¶](#X1084da613df271197e721939747cb602b24d163)

##### "Probing the Importance of Social Structure in Personality State Dynamics: An Exploratory Synthesis of Network Analysis and Machine Learning Modeling"[¶](#X820fedff678b0cdbe484cdbb6787b5e91be9648)

#### Code written by: Damien Lekkas, M.S. (c) 2022[¶](#X047123d64764d36d96bef68945ef042d541e058)

#### Center for Technology and Behavioral Health, Geisel School of Medicine, Dartmouth College, USA[¶](#X89a071bfbba64e78f5780d0d9f0749eb858804a)

#### Program in Quantitative Biomedical Sciences, Dartmouth College, USA[¶](#X978ead306a5e39cb129122c9dc48b1309858daa)

#### STEP 1: IMPORT LIBRARIES AND DEFINE ROOT WORKING DIRECTORY[¶](#Xe9af658d72c49be73780f0c1db46a87a18d62dc)

In [1]:

import networkx as nx
import community
import pandas as pd
import numpy as np
import matplotlib.pyplot as plt
import scipy.stats as stats
from datetime import datetime
import time
import itertools

work_dir = '/users/dlekk/Desktop/IR_network_analysis/'

#### STEP 2: CREATE WEEK-BASED EDGE LISTS[¶](#STEP-2:-CREATE-WEEK-BASED-EDGE-LISTS)

In [2]:

edge_list_ALL = pd.read_csv(work_dir + "Data_IR_withNewLabels.csv", header=0).sort_values(by=["Ego_id", "timestamp", "Alter_id"])
edge_list_ALL['weight'] = [1.0]*edge_list_ALL.shape[0] #add a weights column where each interaction is weighted as 1.0
edge_list_ALL

def edgeSlicer(edge_list_ALL, start_time, end_time):
 date_obj_start = datetime.strptime(start_time, "%m/%d/%Y %H:%M:%S")
 unix_start = int(time.mktime(date_obj_start.timetuple()))*1000000

 date_obj_end = datetime.strptime(end_time, "%m/%d/%Y %H:%M:%S")
 unix_end = int(time.mktime(date_obj_end.timetuple()))*1000000

 edge_list_sliced = edge_list_ALL[(edge_list_ALL['timestamp'] >= unix_start) & (edge_list_ALL['timestamp'] <= unix_end)]

 return edge_list_sliced

#Study weekly start dates
start_times = ["01/30/2012 00:00:00", "02/06/2012 00:00:00", "02/13/2012 00:00:00",
 "02/20/2012 00:00:00", "02/27/2012 00:00:00", "03/05/2012 00:00:00"]

#Study weekly end dates
end_times = ["02/03/2012 23:59:59", "02/10/2012 23:59:59", "02/17/2012 23:59:59",
 "02/24/2012 23:59:59", "03/02/2012 23:59:59", "03/09/2012 23:59:59"]

edge_lists_weekly = []
for week in range(len(start_times)):
 edge_lists_weekly.append(edgeSlicer(edge_list_ALL, start_times[week], end_times[week]))

#### STEP 3: CALCULATE RMSSD FOR EACH PERSONALITY STATE (OUTCOMES FOR MODELING)[¶](#X00c76cba4b6725beb6fa3a5e3298b5bd18594a1)

In [3]:

affect_ALL = pd.read_csv(work_dir + "survey_with_traits_date.csv", header=0).sort_values(by=["ID", "TIMESTAMP"])

def attributeAdder(raw_affect_df, edge_list_df, start_time, end_time):
 affect_ALL = raw_affect_df

 if start_time != None and end_time != None:
 date_obj_start = datetime.strptime(start_time, "%m/%d/%Y %H:%M:%S")
 unix_start = int(time.mktime(date_obj_start.timetuple()))

 date_obj_end = datetime.strptime(end_time, "%m/%d/%Y %H:%M:%S")
 unix_end = int(time.mktime(date_obj_end.timetuple()))

 affect_ALL_sliced = affect_ALL[(affect_ALL['TIMESTAMP'] >= unix_start) & (affect_ALL['TIMESTAMP'] <= unix_end)]
 affect_ALL = affect_ALL_sliced


 affect_ALL_attributes = pd.DataFrame(columns=["Ego_id", "EXTRA_RMSSD", "AGREE_RMSSD", "CONSC_RMSSD", "ESTA_RMSSD", "CREA_RMSSD"])
 for ID in list(set(affect_ALL["ID"])):
 id_df = affect_ALL[affect_ALL["ID"] == ID]
 id_RMSSD_data = [ID]
 for column in list(id_df.columns)[2:]:
 affect_feature = list(id_df[column])
 RMSSD_sum = 0.0
 for i in range(len(affect_feature)-1):
 RMSSD_sum += (affect_feature[i+1] - affect_feature[i])**2
 RMSSD_affect = np.sqrt(RMSSD_sum/id_df.shape[0])
 id_RMSSD_data.append(RMSSD_affect)

 affect_ALL_attributes = affect_ALL_attributes.append({"Ego_id": id_RMSSD_data[0],
 "EXTRA_RMSSD": id_RMSSD_data[1],
 "AGREE_RMSSD": id_RMSSD_data[2],
 "CONSC_RMSSD": id_RMSSD_data[3],
 "ESTA_RMSSD": id_RMSSD_data[4],
 "CREA_RMSSD": id_RMSSD_data[5]}, ignore_index=True)

 affect_ALL_attributes["Ego_id"] = affect_ALL_attributes["Ego_id"].astype(int)
 affect_ALL_attributes = affect_ALL_attributes.sort_values(by=["Ego_id"])

 return affect_ALL_attributes

#Study weekly start dates
start_times = ["01/30/2012 00:00:00", "02/06/2012 00:00:00", "02/13/2012 00:00:00",
 "02/20/2012 00:00:00", "02/27/2012 00:00:00", "03/05/2012 00:00:00"]

#Study weekly end dates
end_times = ["02/03/2012 23:59:59", "02/10/2012 23:59:59", "02/17/2012 23:59:59",
 "02/24/2012 23:59:59", "03/02/2012 23:59:59", "03/09/2012 23:59:59"]

node_attributes_weekly = {}
for week in range(len(start_times)):
 node_attributes_weekly["Week " + str(week+1)] = attributeAdder(affect_ALL, edge_list_ALL, start_times[week], end_times[week])

In [5]:

#Example Output for Week 1 RMSSD personality outcomes across participants
node_attributes_weekly["Week 1"]

Out[5]:

|  | Ego_id | EXTRA_RMSSD | AGREE_RMSSD | CONSC_RMSSD | ESTA_RMSSD | CREA_RMSSD |
| --- | --- | --- | --- | --- | --- | --- |
| 41 | 501 | 0.836660 | 0.570088 | 0.908295 | 0.707107 | 0.741620 |
| 42 | 502 | 0.327327 | 0.707107 | 0.845154 | 0.422577 | 0.462910 |
| 43 | 503 | 1.089725 | 0.381881 | 0.735980 | 0.777282 | 0.735980 |
| 44 | 504 | 1.083974 | 0.591608 | 0.474342 | 0.353553 | 0.670820 |
| 45 | 505 | 1.281740 | 1.260669 | 0.481812 | 0.534522 | 0.767649 |
| 46 | 506 | 2.074505 | 1.802776 | 1.210077 | 2.035401 | 1.546886 |
| 47 | 507 | 1.335415 | 1.125463 | 0.258199 | 0.341565 | 0.408248 |
| 48 | 508 | 1.880350 | 0.755929 | 0.597614 | 1.052209 | 1.149534 |
| 49 | 509 | 2.115420 | 1.313393 | 0.921954 | 0.948683 | 1.565248 |
| 50 | 510 | 1.441153 | 0.843527 | 0.970725 | 0.970725 | 0.796628 |
| 51 | 511 | 1.564106 | 0.566947 | 1.093814 | 0.719623 | 0.876275 |
| 0 | 512 | 1.207615 | 1.607275 | 0.677003 | 1.060660 | 0.790569 |
| 1 | 513 | 1.561249 | 1.425219 | 0.637377 | 0.661438 | 0.790569 |
| 2 | 514 | 0.645497 | 0.612372 | 0.408248 | 0.288675 | 0.577350 |
| 3 | 516 | 1.834498 | 1.037749 | 0.843527 | 0.571772 | 0.416025 |
| 4 | 517 | 1.060660 | 0.612372 | 0.456435 | 0.912871 | 1.136515 |
| 5 | 518 | 0.500000 | 0.540062 | 0.829156 | 0.750000 | 0.353553 |
| 6 | 519 | 0.968246 | 0.500000 | 0.322749 | 1.136515 | 0.677003 |
| 7 | 520 | 2.152816 | 0.832050 | 0.416025 | 0.366900 | 0.796628 |
| 8 | 521 | 1.809068 | 0.753778 | 1.097518 | 1.888963 | 2.158493 |
| 9 | 522 | 1.207615 | 1.010363 | 1.000000 | 1.224745 | 1.030776 |
| 10 | 523 | 1.105542 | 0.408248 | 1.013794 | 0.333333 | 0.745356 |
| 11 | 524 | 3.147871 | 1.566699 | 0.904534 | 1.630672 | 2.667140 |
| 12 | 525 | 0.741620 | 1.024695 | 1.224745 | 1.224745 | 0.223607 |
| 13 | 526 | 1.190238 | 1.207615 | 1.250000 | 1.369306 | 1.040833 |
| 14 | 527 | 1.430194 | 1.167748 | 0.941469 | 1.573935 | 1.128152 |
| 15 | 528 | 1.652504 | 0.746788 | 0.746788 | 0.854850 | 1.184840 |
| 16 | 529 | 0.674200 | 0.866025 | 0.674200 | 0.753778 | 0.583874 |
| 17 | 530 | 0.645497 | 0.288675 | 0.866025 | 0.288675 | 1.290994 |
| 18 | 531 | 1.689428 | 1.118034 | 0.841625 | 1.020621 | 1.443376 |
| 19 | 532 | 2.383656 | 1.348400 | 2.016973 | 1.484771 | 1.758098 |
| 20 | 533 | 1.000000 | 0.547723 | 1.095445 | 0.524404 | 0.670820 |
| 21 | 534 | 0.918559 | 1.060660 | 0.866025 | 0.770552 | 1.346291 |
| 22 | 535 | 1.436141 | 0.204124 | 1.040833 | 0.816497 | 0.577350 |
| 23 | 536 | 1.613743 | 1.020621 | 1.181454 | 0.629153 | 1.299038 |
| 24 | 537 | 0.640870 | 0.640870 | 0.597614 | 0.654654 | 0.707107 |
| 25 | 538 | 1.864135 | 0.474342 | 1.431782 | 1.048809 | 2.207940 |
| 26 | 539 | 1.709701 | 1.652504 | 1.248075 | 0.990338 | 1.000000 |
| 27 | 540 | 0.921954 | 0.341565 | 0.428174 | 0.341565 | 0.846562 |
| 28 | 541 | 0.658281 | 0.129099 | 0.741620 | 0.365148 | 1.032796 |
| 29 | 542 | 0.935414 | 1.354006 | 0.629153 | 1.233221 | 0.829156 |
| 30 | 543 | 0.960769 | 1.176697 | 1.143544 | 0.707107 | 1.176697 |
| 31 | 544 | 1.420094 | 0.763763 | 0.500000 | 0.223607 | 1.161895 |
| 32 | 545 | 0.684653 | 1.758906 | 0.770552 | 1.369306 | 1.391941 |
| 33 | 546 | 0.941469 | 0.301511 | 0.583874 | 0.522233 | 0.941469 |
| 34 | 547 | 1.401530 | 0.906327 | 1.035098 | 0.654654 | 0.744024 |
| 35 | 548 | 1.479020 | 1.154701 | 0.595119 | 0.433013 | 0.540062 |
| 36 | 549 | 1.322876 | 0.801784 | 1.647509 | 0.377964 | 1.141741 |
| 37 | 550 | 1.278019 | 1.870829 | 1.258306 | 1.788854 | 1.016530 |
| 38 | 551 | 0.957427 | 0.645497 | 0.774597 | 1.080123 | 0.826640 |
| 39 | 552 | 0.971825 | 1.632993 | 0.235702 | 0.623610 | 1.054093 |
| 40 | 553 | 2.322893 | 1.172604 | 1.500000 | 0.841625 | 1.547848 |

#### STEP 4: BUILD FUNCTION TO RENDER WEEKLY NETWORKS[¶](#X8361e9c1f4ae5ed18a9398ac877f714ec478e60)

In [6]:

def renderNet(net_dataframe, wt_scale_factor, k, node_color):
 net_ALL_interactions = nx.from_pandas_edgelist(net_dataframe, "Ego_id", "Alter_id", ['weight', 'timestamp'], create_using=nx.MultiDiGraph())

 #Collapse directed multigraph into weighted directed graph
 weight_directed_ALL = nx.DiGraph()
 for u, v, data in net_ALL_interactions.edges(data=True):
 w = data['weight']
 if weight_directed_ALL.has_edge(u,v):
 weight_directed_ALL[u][v]['weight'] += w
 else:
 weight_directed_ALL.add_edge(u, v, weight=w)

 #Authors reported inequivalency in IR detectors flagging interactions in some cases. Since interactions are not
 #directional, we need to simplify this inequivalency. Timestamps is not an option given that even in matched scenarios,
 #the timestamps are not equivalent to the second/millisecond. One option is to take the maximum interaction count between
 #each pair of nodes (e.g., 501 -> 502 and 502 -> 501). This assumes that the malfunctioning IR detector in the interaction
 #is always the same, which may not be true; however, we know in modeling that there had to be at LEAST THAT MANY
 #interactions. With equal treatment across all pairwise nodes, this approach seems reasonable.
 edges_to_drop = []
 completed_edges = []
 for edge in weight_directed_ALL.edges:
 if edge not in completed_edges:
 A = edge[0]
 B = edge[1]
 converse_edge = (B,A)

 if converse_edge in weight_directed_ALL.edges:
 comp = [weight_directed_ALL.edges[(A, B)]['weight'], weight_directed_ALL.edges[(B, A)]['weight']]

 if comp[0] != comp[1]:
 min_index = comp.index(min(comp))
 if min_index == 0:
 drop_edge = (A, B)
 elif min_index == 1:
 drop_edge = (B, A)
 edges_to_drop.append(drop_edge)

 elif comp[0] == comp[1]:
 edges_to_drop.append(converse_edge)

 completed_edges.append(edge)
 completed_edges.append(converse_edge)

 else:
 completed_edges.append(edge)

 edges_to_drop = set(edges_to_drop)

 for etd in edges_to_drop:
 weight_directed_ALL.remove_edge(*etd)

 #Convert now to weighted undirected graph
 weight_undirected_ALL = weight_directed_ALL.to_undirected()

 #Draw graph
 edges = weight_undirected_ALL.edges()
 weights = [weight_undirected_ALL[u][v]['weight']/wt_scale_factor for u,v in edges]

 positioning = nx.spring_layout(weight_undirected_ALL, k=k, iterations=20, seed=10211956)

 fig, ax = plt.subplots(figsize=(10,10))
 netgraph = nx.draw(weight_undirected_ALL,
 positioning,
 alpha=1.0,
 node_size=375,
 node_color=node_color,
 node_shape="o",
 with_labels = True,
 font_size=10,
 font_weight="bold",
 font_color="grey",
 edge_color="black",
 width = weights,
 linewidths=7,
 ax=ax)


 #Function returns weighted undirected network object at index 0
 return weight_undirected_ALL, netgraph

#### STEP 5: BUILD WEEK-BASED SOCIAL NETWORKS OF SUMMATIVE WORKPLACE INTERACTIONS[¶](#X8c57613ee7fb061805f2ff6fd7dfd2eb250bfd1)

##### Week 1 (Jan 30th, 2012 - Feb 3rd, 2012)[¶](#Week-1-(Jan-30th,-2012---Feb-3rd,-2012))

In [7]:

network_week_1 = renderNet(edge_lists_weekly[0], wt_scale_factor=150, k=1.0, node_color="lightskyblue")


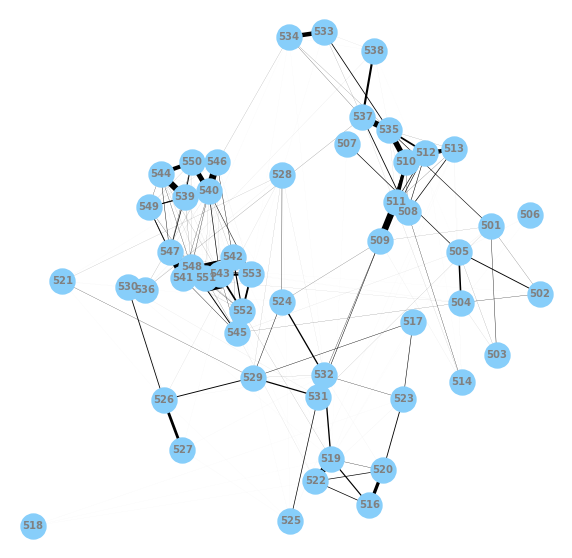


##### Week 2 (Feb 6th, 2012 - Feb 10th, 2012)[¶](#Week-2-(Feb-6th,-2012---Feb-10th,-2012))

In [8]:

network_week_2 = renderNet(edge_lists_weekly[1], wt_scale_factor=150, k=1.0, node_color="lightgreen")


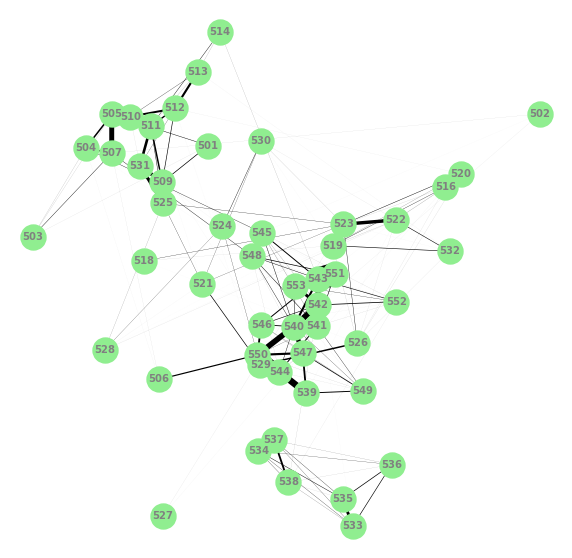


##### Week 3 (Feb 13th, 2012 - Feb 17th, 2012)[¶](#Week-3-(Feb-13th,-2012---Feb-17th,-2012))

In [9]:

network_week_3 = renderNet(edge_lists_weekly[2], wt_scale_factor=150, k=1.0, node_color="khaki")


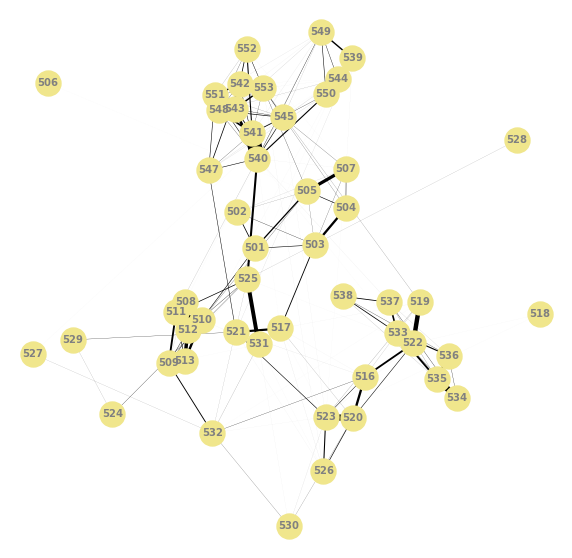


##### Week 4 (Feb 20th, 2012 - Feb 24th, 2012)[¶](#Week-4-(Feb-20th,-2012---Feb-24th,-2012))

In [10]:

network_week_4 = renderNet(edge_lists_weekly[3], wt_scale_factor=150, k=1.0, node_color="lightsalmon")


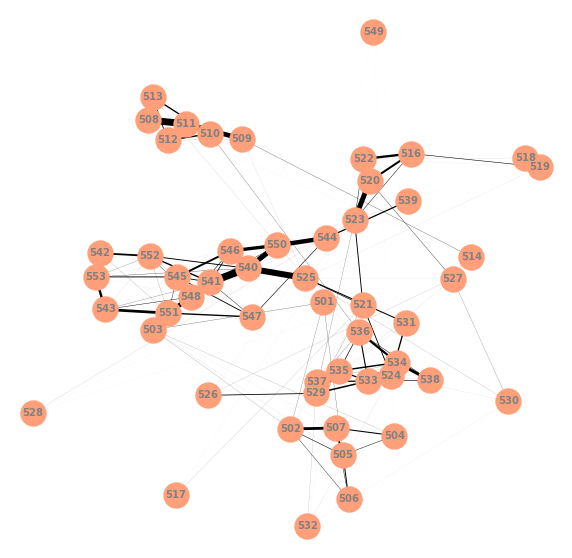


##### Week 5 (Feb 27th, 2012 - Mar 2nd, 2012)[¶](#Week-5-(Feb-27th,-2012---Mar-2nd,-2012))

In [11]:

network_week_5 = renderNet(edge_lists_weekly[4], wt_scale_factor=150, k=1.0, node_color="lightpink")


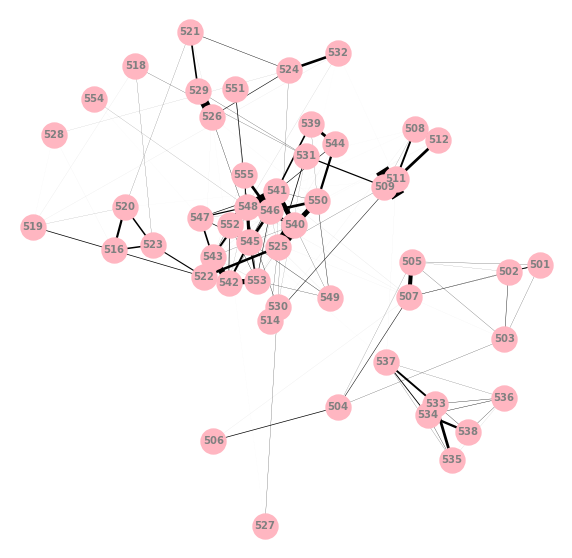


##### Week 6 (Mar 5th, 2012 - Mar 9th, 2012)[¶](#Week-6-(Mar-5th,-2012---Mar-9th,-2012))

In [12]:

network_week_6 = renderNet(edge_lists_weekly[5], wt_scale_factor=150, k=1.0, node_color="thistle")


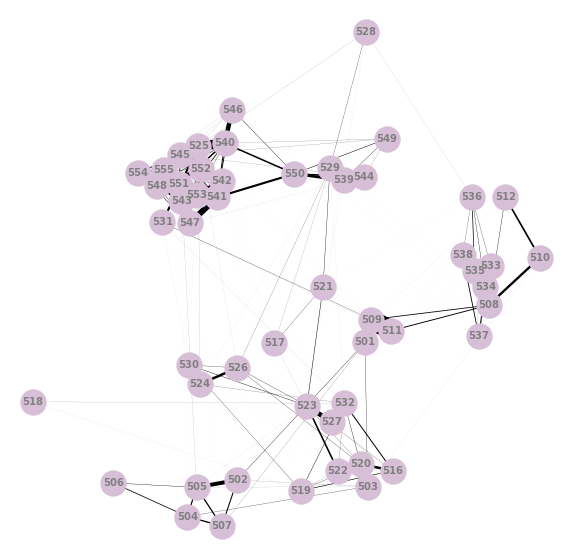


#### STEP 6: COMBINE AND ORGANIZE WEEKLY RMSSD OUTCOME DATA INTO ONE DATAFRAME[¶](#X411b61e49f257437f5bdaff4785a0ffa450eff4)

In [13]:

RMSSD_ATTRIBUTES_WEEKLY = pd.concat([node_attributes_weekly["Week 1"],
 node_attributes_weekly["Week 2"],
 node_attributes_weekly["Week 3"],
 node_attributes_weekly["Week 4"],
 node_attributes_weekly["Week 5"],
 node_attributes_weekly["Week 6"]], axis=0)

RMSSD_ATTRIBUTES_WEEKLY = RMSSD_ATTRIBUTES_WEEKLY.set_index("Ego_id")

In [14]:

#Remove row 115 as there is no corresponding network data for #514 on week 3
RMSSD_ATTRIBUTES_WEEKLY.reset_index(drop=False, inplace=True)
RMSSD_ATTRIBUTES_WEEKLY.drop(RMSSD_ATTRIBUTES_WEEKLY.index[115], inplace=True)
RMSSD_ATTRIBUTES_WEEKLY.reset_index(drop=True, inplace=True)
RMSSD_ATTRIBUTES_WEEKLY.to_csv(work_dir + "rmssd_outcome_attributes_weekly.csv")

In [15]:

#Preview of outcome dataframe
RMSSD_ATTRIBUTES_WEEKLY

Out[15]:

|  | Ego_id | EXTRA_RMSSD | AGREE_RMSSD | CONSC_RMSSD | ESTA_RMSSD | CREA_RMSSD |
| --- | --- | --- | --- | --- | --- | --- |
| 0 | 501 | 0.836660 | 0.570088 | 0.908295 | 0.707107 | 0.741620 |
| 1 | 502 | 0.327327 | 0.707107 | 0.845154 | 0.422577 | 0.462910 |
| 2 | 503 | 1.089725 | 0.381881 | 0.735980 | 0.777282 | 0.735980 |
| 3 | 504 | 1.083974 | 0.591608 | 0.474342 | 0.353553 | 0.670820 |
| 4 | 505 | 1.281740 | 1.260669 | 0.481812 | 0.534522 | 0.767649 |
| ... | ... | ... | ... | ... | ... | ... |
| 302 | 551 | 1.190238 | 1.172604 | 0.612372 | 0.853913 | 0.853913 |
| 303 | 552 | 1.500000 | 1.118034 | 0.000000 | 1.142609 | 0.687184 |
| 304 | 553 | 1.620185 | 0.707107 | 0.612372 | 1.750000 | 1.346291 |
| 305 | 554 | 1.030776 | 1.828592 | 1.346291 | 1.541104 | 0.770552 |
| 306 | 555 | 1.140175 | 1.183216 | 0.387298 | 0.316228 | 0.632456 |

307 rows × 6 columns

#### STEP 7: BUILD FUNCTIONS TO EXTRACT WEEKLY GLOBAL AND EGO NETWORK FEATURES[¶](#Xca8c3dc94da54cae0864e72f60764ee2d28459b)

In [18]:

#Function to construct ego subgraphs
def egoGenerator(network, node):
 #undirected=True builds subgraph of both in and out neighbors instead of just out
 ego_net = nx.ego.ego_graph(network, node, radius=1, center=True, undirected=True, distance="1/weight")

 return ego_net

In [19]:

#Main function (will call egoGenerator)
def networkDeconstructor(network, week):
 #A. NETWORK-BASED FEATURES OF GLOBAL NETWORK
 #1. CALCULATE SIZE OR NUMBER OF DIRECTED TIES
 #Total number of observed connections
 size = network.size()

 #2. CALCULATE ORDER
 #Total number of nodes in the graph
 N = network.order()

 #3. CALCULATE NUMBER OF ORDERED PAIRS
 #Total number of possible directed ties
 #n_directed_ties = N*(N-1)
 n_undirected_ties = (N*(N-1))/2

 #4. CALCULATE CENTRALIZATION
 degrees = dict(network.degree()).values()
 max_deg = max(degrees)
 centralization = float((N*max_deg - sum(degrees)))/(N-1)

 #5. CALCULATE DENSITY (SIZE / NUMBER OF ORDERED PAIRS)
 #How many ties between nodes exist compared to how many ties between nodes are possible
 density = nx.density(network)

 #6. CALCULATE TRANSITIVITY/CLUSTERING COEFFICIENT
 #The fraction of all possible triangles/triads (two nodes with a shared vertex) present
 transitivity = nx.transitivity(network)

 #7. CALCULATE NUMBER OF SUB-COMMUNITIES (LOUVAIN ALGORITHM)
 #Converted to non-directed graph as this is currently not implemented for directed graphs
 #Run algorithm 100 times and return mode
 n_subcommunities = stats.mode([max(community.best_partition(network, partition=None, weight='weight', resolution=1.0, randomize=None, random_state=None).values())+1 for i in range(100)]).mode[0]

 #8. CALCULATE DIAMETER OR MAXIMUM ECCENTRICITY (WEIGHT-AGNOSTIC)
 #The longest path length among all shortest paths from every node to all other nodes
 diameter = nx.diameter(network)

 #9. CALCULATE AVERAGE GEODESIC DISTANCE (ACCOUNTS FOR WEIGHTS)
 geo_dist = nx.average_shortest_path_length(network, weight='1/weight', method="dijkstra")

 network_features = {}
 for node in list(network.nodes):
 network_features[node] = [size,
 n_undirected_ties,
 N,
 centralization,
 density,
 transitivity,
 n_subcommunities,
 diameter,
 geo_dist]

 NETWORK_BASED_FEATURES_DF = pd.DataFrame.from_dict(network_features, orient="index",
 columns=["size",
 "n_undirected_ties",
 "N",
 "centralization",
 "density",
 "transitivity",
 "n_subcommunities",
 "diameter",
 "geo_dist"]).sort_index()

 #B. NODE-BASED FEATURES OF GLOBAL NETWORK
 #1. CALCULATE BETWEENNESS CENTRALITY
 #The percentage of all paths in which a target node is on the path to all other pair-wise nodes
 betw_centrality = pd.DataFrame.from_dict(nx.betweenness_centrality(network, k=None, normalized=True, weight="weight", endpoints=False, seed=10111952), orient="index", columns=["betw_centrality"]).sort_index()

 #2. CALCULATE DEGREE CENTRALITY
 deg_centrality = pd.DataFrame.from_dict(nx.degree_centrality(network), orient="index", columns=["deg_centrality"]).sort_index()

 #3. CALCULATE CLOSENESS CENTRALITY
 #The reciprocal of the sum of the length of the shortest in paths between the node and all other nodes
 closeness_centrality = pd.DataFrame.from_dict(nx.closeness_centrality(network, distance='1/weight'), orient="index", columns=["closeness_centrality"]).sort_index()

 #4. CALCULATE COMMUNITY SIZE
 #community_membership = community.best_partition(nx.Graph(network), partition=None, weight='weight', resolution=1.0, randomize=None, random_state=None)
 community_membership = community.best_partition(network, partition=None, weight='weight', resolution=1.0, randomize=None, random_state=None)

 community_size_percentages = {}
 for i in range(max(list(community_membership.values()))+1):
 community_size_percentages[i] = round(list(community_membership.values()).count(i)/N,3)

 community_data_final = {}
 for i in community_membership.keys():
 community_data_final[i] = community_size_percentages[community_membership[i]]
 community_data_final = pd.DataFrame.from_dict(community_data_final, orient="index", columns=["community_percent_size"]).sort_index()

 NODE_BASED_FEATURES_DF = pd.concat([betw_centrality,
 deg_centrality,
 closeness_centrality,
 community_data_final], axis=1)

 #C. NETWORK-BASED FEATURES OF EGOCENTRIC NETWORK
 EGO_NET_FEATURES_DF = pd.DataFrame(columns=["Ego_id",
 "size_ego",
 "n_undirected_ties_ego",
 "centralization_ego",
 "density_ego",
 "transitivity_ego",
 "diameter_ego",
 "geo_dist_ego",
 "reach_efficiency_ego",
 "betw_centrality_ego",
 "av_dyadic_redundancy_ego",
 "effective_size_ego",
 "efficiency_ego",
 "constraint_ego",
 "av_similarity_ego"])

 for node in list(network.nodes):
 ego_net = egoGenerator(network, node)

 #1. CALCULATE SIZE OR NUMBER OF DIRECTED TIES
 #Total number of observed connections
 size_ego = ego_net.size()

 #2. CALCULATE ORDER
 #Total number of nodes in the graph
 N_ego = ego_net.order()

 #3. CALCULATE NUMBER OF ORDERED PAIRS
 #Total number of possible undirected ties
 n_undirected_ties_ego = (N_ego*(N_ego-1))/2

 #4. CALCULATE CENTRALIZATION
 degrees_ego = dict(ego_net.degree()).values()
 max_deg = max(degrees_ego)
 centralization_ego = float((N*max_deg - sum(degrees_ego)))/(N-1)

 #5. CALCULATE DENSITY (SIZE / NUMBER OF ORDERED PAIRS)
 #How many ties between nodes exist compared to how many ties between nodes are possible
 density_ego = nx.density(ego_net)

 #6. CALCULATE TRANSITIVITY/CLUSTERING COEFFICIENT
 #The fraction of all possible triangles/triads (two nodes with a shared vertex) present
 transitivity_ego = nx.transitivity(ego_net)

 #7. CALCULATE DIAMETER OR MAXIMUM ECCENTRICITY (WEIGHT-AGNOSTIC)
 #The longest path length among all shortest paths from every node to all other nodes
 diameter_ego = nx.diameter(ego_net.to_undirected()) #or max(nx.eccentricity(network.to_undirected()).values())

 #8. CALCULATE AVERAGE GEODESIC DISTANCE (ACCOUNTS FOR WEIGHTS)
 geo_dist_ego = nx.average_shortest_path_length(ego_net, weight='1/weight', method="dijkstra")

 #9. CALCULATE REACH EFFICIENCY OR LOCAL REACHING CENTRALITY OF EGO
 #The proportion of nodes in the network that are within two directed steps of the ego
 reach_efficiency_ego = nx.centrality.local_reaching_centrality(ego_net, node, weight='weight', normalized=True)

 #10. CALCULATE BETWEENNESS CENTRALITY OF EGO
 #The percentage of all paths in which a target node is on the path to all other pair-wise nodes
 betw_centrality_ego = nx.betweenness_centrality(ego_net, k=None, normalized=True, weight="weight", endpoints=False, seed=10111952)[node]

 #11. CALCULATE AVERAGE DYADIC REDUNDANCY
 #The average across alters of how many of the other alters in the neighborhood are also tied to the alter of interest
 ego_alters = list(ego_net.nodes)
 ego_alters.remove(node)

 dyadic_redundancies = []
 for alter in ego_alters:
 if len([x for x in nx.neighbors(ego_net, alter)]) > 1:
 dyadic_redundancies.append(list(nx.bipartite.redundancy.node_redundancy(ego_net, [alter]).values())[0])
 else:
 dyadic_redundancies.append(np.nan)

 av_dyadic_redundancy_ego = np.nanmean(dyadic_redundancies)

 #12. CALCULATE EFFECTIVE SIZE OF EGO IN THEIR EGO NETWORK
 #The number of alters that ego has, minus the average number of ties that each alter has to other alters
 effective_size_ego = nx.structuralholes.effective_size(ego_net, nodes=[node], weight="weight")[node]

 #13. CALCULATE EFFICIENCY OF EGO IN THEIR EGO NETWORK
 #The effective size divided by the degree of the ego
 efficiency_ego = effective_size_ego/ego_net.degree(node)

 #14. CALCULATE CONSTRAINT OF EGO IN THEIR EGO NETWORK
 #The extent to which ego's connections are to others who are connected to one another
 constraint_ego = nx.structuralholes.constraint(ego_net, nodes=[node], weight="weight")[node]

 #15. CALCULATE AVERAGE SIMILARITY (HOMOPHILY)
 #The average number of common neighbors shared by the ego and each of the alters
 common_neighbors = [nx.common_neighbors(ego_net.to_undirected(), node, a) for a in ego_alters]
 total_common_neighbors = []
 for cn in common_neighbors:
 total_common_neighbors.append(len([x for x in cn]))
 av_similarity_ego = np.average(total_common_neighbors)

 EGO_NET_FEATURES_DF = EGO_NET_FEATURES_DF.append({"Ego_id": node,
 "size_ego": size_ego,
 "n_undirected_ties_ego": n_undirected_ties_ego,
 "centralization_ego": centralization_ego,
 "density_ego": density_ego,
 "transitivity_ego": transitivity_ego,
 "diameter_ego": diameter_ego,
 "geo_dist_ego": geo_dist_ego,
 "reach_efficiency_ego": reach_efficiency_ego,
 "betw_centrality_ego": betw_centrality_ego,
 "av_dyadic_redundancy_ego": av_dyadic_redundancy_ego,
 "effective_size_ego": effective_size_ego,
 "efficiency_ego": efficiency_ego,
 "constraint_ego": constraint_ego,
 "av_similarity_ego": av_similarity_ego}, ignore_index=True)

 EGO_NET_FEATURES_DF['Ego_id'] = EGO_NET_FEATURES_DF['Ego_id'].astype(int)
 EGO_NET_FEATURES_DF = EGO_NET_FEATURES_DF.set_index('Ego_id').sort_index()


 #Put all dataframes together
 ALL_FEATURES_DF = pd.concat([NETWORK_BASED_FEATURES_DF, NODE_BASED_FEATURES_DF, EGO_NET_FEATURES_DF], axis=1)
 ALL_FEATURES_DF['Week'] = [week]*ALL_FEATURES_DF.shape[0]

 return ALL_FEATURES_DF

#### STEP 8: CREATE EXTRACTED WEEKLY NETWORK FEATURE DATAFRAME[¶](#Xbfd347ed36b3273eb1053acb908f541138b0a7e)

In [21]:

NETWORK_FEATURE_SPACE_WEEKLY = pd.concat([networkDeconstructor(network_week_1[0], 1),
 networkDeconstructor(network_week_2[0], 2),
 networkDeconstructor(network_week_3[0], 3),
 networkDeconstructor(network_week_4[0], 4),
 networkDeconstructor(network_week_5[0], 5),
 networkDeconstructor(network_week_6[0], 6)], axis=0)

NETWORK_FEATURE_SPACE_WEEKLY.reset_index(drop=False, inplace=True)
NETWORK_FEATURE_SPACE_WEEKLY.rename(columns={'index': "Ego_id"}, inplace=True)

<ipython-input-19-c4bf0bfb8727>:167: RuntimeWarning: Mean of empty slice
 av_dyadic_redundancy_ego = np.nanmean(dyadic_redundancies)

In [22]:

#Preview of feature dataframe
NETWORK_FEATURE_SPACE_WEEKLY

[TABLE PREVIEW REMOVED]

307 rows × 29 columns

#### STEP 9: COMBINE NETWORK FEATURE DATAFRAME WITH RMSSD OUTCOME DATAFRAME[¶](#X1e9045370642dafc7eac32d79cafa01a1eeee8d)

In [23]:

FINAL_DATA_FRAME_WEEKLY = pd.concat([NETWORK_FEATURE_SPACE_WEEKLY, RMSSD_ATTRIBUTES_WEEKLY], axis=1)
FINAL_DATA_FRAME_WEEKLY

Out[23]:

[TABLE PREVIEW REMOVED]

307 rows × 35 columns

In [28]:

#Write final dataframe to csv for machine learning modeling in R
FINAL_DATA_FRAME_WEEKLY.to_csv(work_dir + "DATA_FINAL_WEEKLY.csv")
