# Supplementary material for: Using passive sensor data to probe associations of social structure with changes in personality: A synthesis of network analysis and machine learning
Source: PLoS One. 2022 Nov 30;17(11):e0277516. doi: 10.1371/journal.pone.0277516 (PMC9710841; doi:10.1371/journal.pone.0277516)
Supplement: S3 File — (DOCX) [file pone.0277516.s003.docx]

ML Code for Network Personality Dynamics

Damien Lekkas

June 2022

STEP 0: READ IN AND WITHIN-FEATURE STANDARDIZE DATA

work_dir = '/users/dlekk/Desktop/IR_network_analysis/'
data_in <- read.csv(paste(work_dir, "DATA_FINAL_WEEKLY.csv", sep=""), header=TRUE)
data_in$diameter_ego <- NULL

#CHANGE NA IN AVERAGE DYADIC REDUNDANCY TO -1
data_in$av_dyadic_redundancy_ego[which(is.na(data_in$av_dyadic_redundancy_ego))] <- -1

#WITHIN-FEATURE STANDARDIZE
data_std = cbind(data_in[,1:2], apply(data_in[,3:28], 2, function(x) scale(x)), data_in[,29:33])

***MODEL 1: EXTRAVERSION***

STEP 1: BUILD AND RUN MODEL

library(caret)
library(xgboost)
library(boot)

#NUMBER OF SUBJECTS (N=54)
N <- length(unique(data_std$Ego_id))

#INITIATE LIST OBJECTS TO HOLD FOLD-BASED ROW INDICES BELONGING TO PERTINENT SUBJECTS
#MOST SUBJECTS ARE REPRESENTED 6 TIMES IN THE DATASET BECAUSE THERE ARE RMSSD OUTCOME VALUES FOR 6 SEPARATE WEEKS
list_of_training_idxs <- list()
list_of_validation_idxs <- list()

i <- 1
for (eid in unique(data_std$Ego_id)) {
 training_idxs <- which(data_std$Ego_id != eid)
 list_of_training_idxs[[i]] <- training_idxs

 validation_indices <- which(data_std$Ego_id == eid)
 list_of_validation_idxs[[i]] <- validation_indices
 i <- i + 1
 }

#REMOVE EGO_ID AND UNUSED OUTCOME FEATURES
test_data <- data_std[,-c(1,30,31,32,33)]

#RESET ROWNAMES
rownames(test_data) <- NULL

#SET TRAIN CONTROL VARIABLE AND SPECIFY ROW INDICES OF TRAINING DATA TO USE
set.seed(10111952)
seeds_1 <- vector(mode="list", length=55)
for(i in 1:54) seeds_1[[i]] <- sample.int(10000,36)
seeds_1[[55]] <- sample.int(10000,1)

fitControl_1 <- trainControl(method = "cv", #Note CV use set to N-1 folds; equivalent to LOOCV
 number = N-1, #Set folds
 savePredictions = TRUE,
 index = list_of_training_idxs, #Define row indices for each fold
 seeds = seeds_1)

#RUN TREE-BASED BOOSTING ENSEMBLE MODEL AND OPTIMIZE FOR R-SQUARED (% VARIANCE EXPLAINED)
model_out_1 <- train(EXTRA_RMSSD ~ ., data = test_data,
 method = 'xgbTree',
 metric = "Rsquared",
 trControl = fitControl_1
 )

#SAVE BEST-TUNED MODEL RESULTS
model_out_1_results <- data.frame(model_out_1$results[rownames(model_out_1$bestTune),])

#EXTRACT PREDICTIONS OF BEST-TUNED MODEL
m1_fold_predictions <- matrix(nrow=dim(data_in)[1], ncol=2)
m1_preds <- model_out_1$pred[which(model_out_1$pred$nrounds == model_out_1$bestTune$nrounds &
 model_out_1$pred$max_depth == model_out_1$bestTune$max_depth &
 model_out_1$pred$gamma == model_out_1$bestTune$gamma &
 model_out_1$pred$min_child_weight == model_out_1$bestTune$min_child_weight &
 model_out_1$pred$eta == model_out_1$bestTune$eta &
 model_out_1$pred$subsample == model_out_1$bestTune$subsample &
 model_out_1$pred$colsample_bytree == model_out_1$bestTune$colsample_bytree),]

#CREATE DATAFRAME TO STORE MODEL PREDICTIONS (COL 1) AND OBSERVED OUTCOME VALUES (COL 2)
for (row in (1:dim(data_in)[1])) {
 row_data <- m1_preds[which(m1_preds$rowIndex==row),]
 m1_fold_predictions[row,1] <- row_data$pred
 m1_fold_predictions[row,2] <- row_data$obs
}

model_out_1_results

STEP 2: SAVE IN-FOLD (SUBJECT-LEVEL) MODEL 1 PERFORMANCE

m1_resamples <- model_out_1$resample
m1_resamples_filtered <- m1_resamples[which(m1_resamples$Rsquared != 1.0),] #remove subjects with only two data points

par(mar = c(5, 5, 3, 5))
hist(m1_resamples_filtered$Rsquared, prob=FALSE, col="grey", breaks = 10, ylab="Count", xlab="R-squared", main="")

par(new=TRUE)
plot(density(m1_resamples_filtered$Rsquared), col="blue", lwd=2, xaxt="n", yaxt="n", main="", xlab="", ylab="", xlim=c(0,1))
axis(side=4)
mtext("Density", side=4, line=3)

STEP 3: CALCULATE MODEL 1 VARIABLE IMPORTANCE

(imp_1 <- varImp(model_out_1, scale=TRUE))

STEP 4: CREATE COWPLOTS TO ILLUSTRATE VARIABILITY IN PERFORMANCE ACROSS INDIVIDUALS

library(cowplot)

s <- 501
i <- 1
plot_list <- c()
for (val_list in 1:length(list_of_validation_idxs)) {
 subj_preds <- m1_preds[which(m1_preds$rowIndex %in% list_of_validation_idxs[[val_list]]),]$pred
 subj_obs <- m1_preds[which(m1_preds$rowIndex %in% list_of_validation_idxs[[val_list]]),]$obs
 subj_data <- data.frame(cbind(SUBJ_OBS=subj_obs, SUBJ_PREDS=subj_preds))

 if (s < 515) {
 p <- ggplot(subj_data, aes(x=SUBJ_OBS, y=SUBJ_PREDS)) + geom_point() +
 geom_abline(linetype = "dashed", color="blue") +
 geom_text(label= paste("R2 = ", round(m1_resamples$Rsquared[which(m1_resamples$Resample == paste("Resample", substr(as.character(s), 2,3), sep=""))],2), sep=""), x=0.25, y=3.75, size=2) +
 xlim(c(0,4.0)) +
 ylim(c(0,4.0)) +
 ggtitle(as.character(s)) +
 theme(plot.title = element_text(hjust = 0.5, size=10)) +
 labs(x="", y="")
 }
 else if (s == 515){
 s <- 516
 p <- ggplot(subj_data, aes(x=SUBJ_OBS, y=SUBJ_PREDS)) + geom_point() +
 geom_abline(linetype = "dashed", color="blue") +
 geom_text(label= paste("R2 = ", round(m1_resamples$Rsquared[which(m1_resamples$Resample == paste("Resample", substr(as.character(s-1), 2,3), sep=""))],2),sep=""), x=0.25, y=3.75, size=2) +
 xlim(c(0,4.0)) +
 ylim(c(0,4.0)) +
 ggtitle(as.character(s)) +
 theme(plot.title = element_text(hjust = 0.5, size=10)) +
 labs(x="", y="")

 }
 else if (s > 515){
 p <- ggplot(subj_data, aes(x=SUBJ_OBS, y=SUBJ_PREDS)) + geom_point() +
 geom_abline(linetype = "dashed", color="blue") +
 geom_text(label= paste("R2 = ", round(m1_resamples$Rsquared[which(m1_resamples$Resample == paste("Resample", substr(as.character(s-1), 2,3), sep=""))],2), sep=""), x=0.25, y=3.75, size=2) +
 xlim(c(0,4.0)) +
 ylim(c(0,4.0)) +
 ggtitle(as.character(s)) +
 theme(plot.title = element_text(hjust = 0.5, size=10)) +
 labs(x="", y="")

 }

 plot_list[[i]] <- p
 i <- i + 1
 s <- s + 1
 }

(pg_1A <- plot_grid(plotlist=plot_list[1:9], ncol=3))
(pg_2A <- plot_grid(plotlist=plot_list[10:18], ncol=3))
(pg_3A <- plot_grid(plotlist=plot_list[19:27], ncol=3))
(pg_4A <- plot_grid(plotlist=plot_list[28:36], ncol=3))
(pg_5A <- plot_grid(plotlist=plot_list[37:45], ncol=3))
(pg_6A <- plot_grid(plotlist=plot_list[46:54], ncol=3))

***MODEL 2: AGREEABLENESS***

STEP 1: BUILD AND RUN MODEL

library(caret)
library(xgboost)
library(boot)

#NUMBER OF SUBJECTS (N=54)
N <- length(unique(data_std$Ego_id))

#INITIATE LIST OBJECTS TO HOLD FOLD-BASED ROW INDICES BELONGING TO PERTINENT SUBJECTS
#MOST SUBJECTS ARE REPRESENTED 6 TIMES IN THE DATASET BECAUSE THERE ARE RMSSD OUTCOME VALUES FOR 6 SEPARATE WEEKS
list_of_training_idxs <- list()
list_of_validation_idxs <- list()

i <- 1
for (eid in unique(data_std$Ego_id)) {
 training_idxs <- which(data_std$Ego_id != eid)
 list_of_training_idxs[[i]] <- training_idxs

 validation_indices <- which(data_std$Ego_id == eid)
 list_of_validation_idxs[[i]] <- validation_indices
 i <- i + 1
 }

#REMOVE EGO_ID AND UNUSED OUTCOME FEATURES
test_data <- data_std[,-c(1,29,31,32,33)]

#RESET ROWNAMES TO AVOID SECRET SHENANIGANS
rownames(test_data) <- NULL

#SET TRAIN CONTROL VARIABLE AND SPECIFY ROW INDICES OF TRAINING DATA TO USE
set.seed(10111952)
seeds_2 <- vector(mode="list", length=55)
for(i in 1:54) seeds_2[[i]] <- sample.int(10000,36)
seeds_2[[55]] <- sample.int(10000,1)

fitControl_2 <- trainControl(method = "cv", #Note CV use set to N-1 folds; equivalent to LOOCV
 number = N-1, #Set folds
 savePredictions = TRUE,
 index = list_of_training_idxs, #Define row indices for each fold
 seeds = seeds_2)

#RUN TREE-BASED BOOSTING ENSEMBLE MODEL AND OPTIMIZE FOR R-SQUARED (% VARIANCE EXPLAINED)
model_out_2 <- train(AGREE_RMSSD ~ ., data = test_data,
 method = 'xgbTree',
 metric = "Rsquared",
 trControl = fitControl_2
 )

#SAVE BEST-TUNED MODEL RESULTS
model_out_2_results <- data.frame(model_out_2$results[rownames(model_out_2$bestTune),])

#EXTRACT PREDICTIONS OF BEST-TUNED MODEL
m2_fold_predictions <- matrix(nrow=dim(data_in)[1], ncol=2)

m2_preds <- model_out_2$pred[which(model_out_2$pred$nrounds == model_out_2$bestTune$nrounds &
 model_out_2$pred$max_depth == model_out_2$bestTune$max_depth &
 model_out_2$pred$gamma == model_out_2$bestTune$gamma &
 model_out_2$pred$min_child_weight == model_out_2$bestTune$min_child_weight &
 model_out_2$pred$eta == model_out_2$bestTune$eta &
 model_out_2$pred$subsample == model_out_2$bestTune$subsample &
 model_out_2$pred$colsample_bytree == model_out_2$bestTune$colsample_bytree),]

#CREATE DATAFRAME TO STORE MODEL PREDICTIONS (COL 1) AND OBSERVED OUTCOME VALUES (COL 2)
for (row in (1:dim(data_in)[1])) {
 row_data <- m2_preds[which(m1_preds$rowIndex==row),]
 m2_fold_predictions[row,1] <- row_data$pred
 m2_fold_predictions[row,2] <- row_data$obs
}

model_out_2_results

STEP 2: SAVE IN-FOLD (SUBJECT-LEVEL) MODEL 2 PERFORMANCE

m2_resamples <- model_out_2$resample
m2_resamples_filtered <- m2_resamples[which(m2_resamples$Rsquared != 1.0),] #remove subjects with only two data points

par(mar = c(5, 5, 3, 5))
hist(m2_resamples_filtered$Rsquared, prob=FALSE, col="grey", breaks = 10, ylab="Count", xlab="R-squared", main="")

par(new=TRUE)
plot(density(m2_resamples_filtered$Rsquared), col="blue", lwd=2, xaxt="n", yaxt="n", main="", xlab="", ylab="", xlim=c(0,1))
axis(side=4)
mtext("Density", side=4, line=3)

STEP 3: CALCULATE MODEL 2 VARIABLE IMPORTANCE

(imp_2 <- varImp(model_out_2, scale=TRUE))

STEP 4: CREATE COWPLOTS TO ILLUSTRATE VARIABILITY IN PERFORMANCE ACROSS INDIVIDUALS

library(cowplot)

s <- 501
i <- 1
plot_list <- c()
for (val_list in 1:length(list_of_validation_idxs)) {
 subj_preds <- m2_preds[which(m2_preds$rowIndex %in% list_of_validation_idxs[[val_list]]),]$pred
 subj_obs <- m2_preds[which(m2_preds$rowIndex %in% list_of_validation_idxs[[val_list]]),]$obs
 subj_data <- data.frame(cbind(SUBJ_OBS=subj_obs, SUBJ_PREDS=subj_preds))

 if (s < 515) {
 p <- ggplot(subj_data, aes(x=SUBJ_OBS, y=SUBJ_PREDS)) + geom_point() +
 geom_abline(linetype = "dashed", color="blue") +
 geom_text(label= paste("R2 = ", round(m2_resamples$Rsquared[which(m2_resamples$Resample == paste("Resample", substr(as.character(s), 2,3), sep=""))],2), sep=""), x=0.25, y=3.75, size=2) +
 xlim(c(0,4.0)) +
 ylim(c(0,4.0)) +
 ggtitle(as.character(s)) +
 theme(plot.title = element_text(hjust = 0.5, size=10)) +
 labs(x="", y="")
 }
 else if (s == 515){
 s <- 516
 p <- ggplot(subj_data, aes(x=SUBJ_OBS, y=SUBJ_PREDS)) + geom_point() +
 geom_abline(linetype = "dashed", color="blue") +
 geom_text(label= paste("R2 = ", round(m2_resamples$Rsquared[which(m2_resamples$Resample == paste("Resample", substr(as.character(s-1), 2,3), sep=""))],2),sep=""), x=0.25, y=3.75, size=2) +
 xlim(c(0,4.0)) +
 ylim(c(0,4.0)) +
 ggtitle(as.character(s)) +
 theme(plot.title = element_text(hjust = 0.5, size=10)) +
 labs(x="", y="")

 }
 else if (s > 515){
 p <- ggplot(subj_data, aes(x=SUBJ_OBS, y=SUBJ_PREDS)) + geom_point() +
 geom_abline(linetype = "dashed", color="blue") +
 geom_text(label= paste("R2 = ", round(m2_resamples$Rsquared[which(m2_resamples$Resample == paste("Resample", substr(as.character(s-1), 2,3), sep=""))],2), sep=""), x=0.25, y=3.75, size=2) +
 xlim(c(0,4.0)) +
 ylim(c(0,4.0)) +
 ggtitle(as.character(s)) +
 theme(plot.title = element_text(hjust = 0.5, size=10)) +
 labs(x="", y="")

 }

 plot_list[[i]] <- p
 i <- i + 1
 s <- s + 1
 }

(pg_1B <- plot_grid(plotlist=plot_list[1:9], ncol=3))
(pg_2B <- plot_grid(plotlist=plot_list[10:18], ncol=3))
(pg_3B <- plot_grid(plotlist=plot_list[19:27], ncol=3))
(pg_4B <- plot_grid(plotlist=plot_list[28:36], ncol=3))
(pg_5B <- plot_grid(plotlist=plot_list[37:45], ncol=3))
(pg_6B <- plot_grid(plotlist=plot_list[46:54], ncol=3))

***MODEL 3: CONSCIENTIOUSNESS***

STEP 1: BUILD AND RUN MODEL

library(caret)
library(xgboost)
library(boot)

#NUMBER OF SUBJECTS (N=54)
N <- length(unique(data_std$Ego_id))

#INITIATE LIST OBJECTS TO HOLD FOLD-BASED ROW INDICES BELONGING TO PERTINENT SUBJECTS
#MOST SUBJECTS ARE REPRESENTED 6 TIMES IN THE DATASET BECAUSE THERE ARE RMSSD OUTCOME VALUES FOR 6 SEPARATE WEEKS
list_of_training_idxs <- list()
list_of_validation_idxs <- list()

i <- 1
for (eid in unique(data_std$Ego_id)) {
 training_idxs <- which(data_std$Ego_id != eid)
 list_of_training_idxs[[i]] <- training_idxs

 validation_indices <- which(data_std$Ego_id == eid)
 list_of_validation_idxs[[i]] <- validation_indices
 i <- i + 1
 }

#REMOVE EGO_ID AND UNUSED OUTCOME FEATURES
test_data <- data_std[,-c(1,29,30,32,33)]


#RESET ROWNAMES TO AVOID SECRET SHENANIGANS
rownames(test_data) <- NULL

#SET TRAIN CONTROL VARIABLE AND SPECIFY ROW INDICES OF TRAINING DATA TO USE
set.seed(10111952)
seeds_3 <- vector(mode="list", length=55)
for(i in 1:54) seeds_3[[i]] <- sample.int(10000,36)
seeds_3[[55]] <- sample.int(10000,1)

fitControl_3 <- trainControl(method = "cv", #Note CV use set to N-1 folds; equivalent to LOOCV
 number = N-1, #Set folds
 savePredictions = TRUE,
 index = list_of_training_idxs, #Define row indices for each fold
 seeds = seeds_3)

#RUN TREE-BASED BOOSTING ENSEMBLE MODEL AND OPTIMIZE FOR R-SQUARED (% VARIANCE EXPLAINED)
model_out_3 <- train(CONSC_RMSSD ~ ., data = test_data,
 method = 'xgbTree',
 metric = "Rsquared",
 trControl = fitControl_3
 )

#SAVE BEST-TUNED MODEL RESULTS
model_out_3_results <- data.frame(model_out_3$results[rownames(model_out_3$bestTune),])

#EXTRACT PREDICTIONS OF BEST-TUNED MODEL
m3_fold_predictions <- matrix(nrow=dim(data_in)[1], ncol=2)

m3_preds <- model_out_3$pred[which(model_out_3$pred$nrounds == model_out_3$bestTune$nrounds &
 model_out_3$pred$max_depth == model_out_3$bestTune$max_depth &
 model_out_3$pred$gamma == model_out_3$bestTune$gamma &
 model_out_3$pred$min_child_weight == model_out_3$bestTune$min_child_weight &
 model_out_3$pred$eta == model_out_3$bestTune$eta &
 model_out_3$pred$subsample == model_out_3$bestTune$subsample &
 model_out_3$pred$colsample_bytree == model_out_3$bestTune$colsample_bytree),]

#CREATE DATAFRAME TO STORE MODEL PREDICTIONS (COL 1) AND OBSERVED OUTCOME VALUES (COL 2)
for (row in (1:dim(data_in)[1])) {
 row_data <- m3_preds[which(m3_preds$rowIndex==row),]
 m3_fold_predictions[row,1] <- row_data$pred
 m3_fold_predictions[row,2] <- row_data$obs
}

model_out_3_results

STEP 2: SAVE IN-FOLD (SUBJECT-LEVEL) MODEL 3 PERFORMANCE

m3_resamples <- model_out_3$resample
m3_resamples_filtered <- m3_resamples[which(m3_resamples$Rsquared != 1.0),] #remove subjects with only two data points

par(mar = c(5, 5, 3, 5))
hist(m3_resamples_filtered$Rsquared, prob=FALSE, col="grey", breaks = 10, ylab="Count", xlab="R-squared", main="")

par(new=TRUE)
plot(density(m3_resamples_filtered$Rsquared), col="blue", lwd=2, xaxt="n", yaxt="n", main="", xlab="", ylab="", xlim=c(0,1))
axis(side=4)
mtext("Density", side=4, line=3)

STEP 3: CALCULATE MODEL 3 VARIABLE IMPORTANCE

(imp_3 <- varImp(model_out_3, scale=TRUE))

STEP 4: CREATE COWPLOTS TO ILLUSTRATE VARIABILITY IN PERFORMANCE ACROSS INDIVIDUALS

library(cowplot)

s <- 501
i <- 1
plot_list <- c()
for (val_list in 1:length(list_of_validation_idxs)) {
 subj_preds <- m3_preds[which(m3_preds$rowIndex %in% list_of_validation_idxs[[val_list]]),]$pred
 subj_obs <- m3_preds[which(m3_preds$rowIndex %in% list_of_validation_idxs[[val_list]]),]$obs
 subj_data <- data.frame(cbind(SUBJ_OBS=subj_obs, SUBJ_PREDS=subj_preds))

 if (s < 515) {
 p <- ggplot(subj_data, aes(x=SUBJ_OBS, y=SUBJ_PREDS)) + geom_point() +
 geom_abline(linetype = "dashed", color="blue") +
 geom_text(label= paste("R2 = ", round(m3_resamples$Rsquared[which(m3_resamples$Resample == paste("Resample", substr(as.character(s), 2,3), sep=""))],2), sep=""), x=0.25, y=3.75, size=2) +
 xlim(c(0,4.0)) +
 ylim(c(0,4.0)) +
 ggtitle(as.character(s)) +
 theme(plot.title = element_text(hjust = 0.5, size=10)) +
 labs(x="", y="")
 }
 else if (s == 515){
 s <- 516
 p <- ggplot(subj_data, aes(x=SUBJ_OBS, y=SUBJ_PREDS)) + geom_point() +
 geom_abline(linetype = "dashed", color="blue") +
 geom_text(label= paste("R2 = ", round(m3_resamples$Rsquared[which(m3_resamples$Resample == paste("Resample", substr(as.character(s-1), 2,3), sep=""))],2),sep=""), x=0.25, y=3.75, size=2) +
 xlim(c(0,4.0)) +
 ylim(c(0,4.0)) +
 ggtitle(as.character(s)) +
 theme(plot.title = element_text(hjust = 0.5, size=10)) +
 labs(x="", y="")

 }
 else if (s > 515){
 p <- ggplot(subj_data, aes(x=SUBJ_OBS, y=SUBJ_PREDS)) + geom_point() +
 geom_abline(linetype = "dashed", color="blue") +
 geom_text(label= paste("R2 = ", round(m3_resamples$Rsquared[which(m3_resamples$Resample == paste("Resample", substr(as.character(s-1), 2,3), sep=""))],2), sep=""), x=0.25, y=3.75, size=2) +
 xlim(c(0,4.0)) +
 ylim(c(0,4.0)) +
 ggtitle(as.character(s)) +
 theme(plot.title = element_text(hjust = 0.5, size=10)) +
 labs(x="", y="")

 }

 plot_list[[i]] <- p
 i <- i + 1
 s <- s + 1
 }

(pg_1C <- plot_grid(plotlist=plot_list[1:9], ncol=3))
(pg_2C <- plot_grid(plotlist=plot_list[10:18], ncol=3))
(pg_3C <- plot_grid(plotlist=plot_list[19:27], ncol=3))
(pg_4C <- plot_grid(plotlist=plot_list[28:36], ncol=3))
(pg_5C <- plot_grid(plotlist=plot_list[37:45], ncol=3))
(pg_6C <- plot_grid(plotlist=plot_list[46:54], ncol=3))

***MODEL 4: EMOTIONAL STABILITY***

STEP 1: BUILD AND RUN MODEL

library(caret)
library(xgboost)
library(boot)

#NUMBER OF SUBJECTS (N=54)
N <- length(unique(data_std$Ego_id))

#INITIATE LIST OBJECTS TO HOLD FOLD-BASED ROW INDICES BELONGING TO PERTINENT SUBJECTS
#MOST SUBJECTS ARE REPRESENTED 6 TIMES IN THE DATASET BECAUSE THERE ARE RMSSD OUTCOME VALUES FOR 6 SEPARATE WEEKS
list_of_training_idxs <- list()
list_of_validation_idxs <- list()

i <- 1
for (eid in unique(data_std$Ego_id)) {
 training_idxs <- which(data_std$Ego_id != eid)
 list_of_training_idxs[[i]] <- training_idxs

 validation_indices <- which(data_std$Ego_id == eid)
 list_of_validation_idxs[[i]] <- validation_indices
 i <- i + 1
 }

#REMOVE EGO_ID AND UNUSED OUTCOME FEATURES
test_data <- data_std[,-c(1,29,30,31,33)]

#RESET ROWNAMES TO AVOID SECRET SHENANIGANS
rownames(test_data) <- NULL

#SET TRAIN CONTROL VARIABLE AND SPECIFY ROW INDICES OF TRAINING DATA TO USE
set.seed(10111952)
seeds_4 <- vector(mode="list", length=55)
for(i in 1:54) seeds_4[[i]] <- sample.int(10000,36)
seeds_4[[55]] <- sample.int(10000,1)

fitControl_4 <- trainControl(method = "cv", #Note CV use set to N-1 folds; equivalent to LOOCV
 number = N-1, #Set folds
 savePredictions = TRUE,
 index = list_of_training_idxs, #Define row indices for each fold
 seeds = seeds_4)

#RUN TREE-BASED BOOSTING ENSEMBLE MODEL AND OPTIMIZE FOR R-SQUARED (% VARIANCE EXPLAINED)
model_out_4 <- train(ESTA_RMSSD ~ ., data = test_data,
 method = 'xgbTree',
 metric = "Rsquared",
 trControl = fitControl_4)

#SAVE BEST-TUNED MODEL RESULTS
model_out_4_results <- data.frame(model_out_4$results[rownames(model_out_4$bestTune),])

#EXTRACT PREDICTIONS OF BEST-TUNED MODEL
m4_fold_predictions <- matrix(nrow=dim(data_in)[1], ncol=2)

m4_preds <- model_out_4$pred[which(model_out_4$pred$nrounds == model_out_4$bestTune$nrounds &
 model_out_4$pred$max_depth == model_out_4$bestTune$max_depth &
 model_out_4$pred$gamma == model_out_4$bestTune$gamma &
 model_out_4$pred$min_child_weight == model_out_4$bestTune$min_child_weight &
 model_out_4$pred$eta == model_out_4$bestTune$eta &
 model_out_4$pred$subsample == model_out_4$bestTune$subsample &
 model_out_4$pred$colsample_bytree == model_out_4$bestTune$colsample_bytree),]

#CREATE DATAFRAME TO STORE MODEL PREDICTIONS (COL 1) AND OBSERVED OUTCOME VALUES (COL 2)
for (row in (1:dim(data_in)[1])) {
 row_data <- m4_preds[which(m4_preds$rowIndex==row),]
 m4_fold_predictions[row,1] <- row_data$pred
 m4_fold_predictions[row,2] <- row_data$obs
}

model_out_4_results

STEP 2: SAVE IN-FOLD (SUBJECT-LEVEL) MODEL 4 PERFORMANCE

m4_resamples <- model_out_4$resample
m4_resamples_filtered <- m4_resamples[which(m4_resamples$Rsquared != 1.0),] #remove subjects with only two data points

par(mar = c(5, 5, 3, 5))
hist(m4_resamples_filtered$Rsquared, prob=FALSE, col="grey", breaks = 10, ylab="Count", xlab="R-squared", main="")

par(new=TRUE)
plot(density(m4_resamples_filtered$Rsquared), col="blue", lwd=2, xaxt="n", yaxt="n", main="", xlab="", ylab="", xlim=c(0,1))
axis(side=4)
mtext("Density", side=4, line=3)

STEP 3: CALCULATE MODEL 4 VARIABLE IMPORTANCE

(imp_4 <- varImp(model_out_4, scale=TRUE))

STEP 4: CREATE COWPLOTS TO ILLUSTRATE VARIABILITY IN PERFORMANCE ACROSS INDIVIDUALS

library(cowplot)

s <- 501
i <- 1
plot_list <- c()
for (val_list in 1:length(list_of_validation_idxs)) {
 subj_preds <- m4_preds[which(m4_preds$rowIndex %in% list_of_validation_idxs[[val_list]]),]$pred
 subj_obs <- m4_preds[which(m4_preds$rowIndex %in% list_of_validation_idxs[[val_list]]),]$obs
 subj_data <- data.frame(cbind(SUBJ_OBS=subj_obs, SUBJ_PREDS=subj_preds))

 if (s < 515) {
 p <- ggplot(subj_data, aes(x=SUBJ_OBS, y=SUBJ_PREDS)) + geom_point() +
 geom_abline(linetype = "dashed", color="blue") +
 geom_text(label= paste("R2 = ", round(m4_resamples$Rsquared[which(m4_resamples$Resample == paste("Resample", substr(as.character(s), 2,3), sep=""))],2), sep=""), x=0.25, y=3.75, size=2) +
 xlim(c(0,4.0)) +
 ylim(c(0,4.0)) +
 ggtitle(as.character(s)) +
 theme(plot.title = element_text(hjust = 0.5, size=10)) +
 labs(x="", y="")
 }
 else if (s == 515){
 s <- 516
 p <- ggplot(subj_data, aes(x=SUBJ_OBS, y=SUBJ_PREDS)) + geom_point() +
 geom_abline(linetype = "dashed", color="blue") +
 geom_text(label= paste("R2 = ", round(m4_resamples$Rsquared[which(m4_resamples$Resample == paste("Resample", substr(as.character(s-1), 2,3), sep=""))],2),sep=""), x=0.25, y=3.75, size=2) +
 xlim(c(0,4.0)) +
 ylim(c(0,4.0)) +
 ggtitle(as.character(s)) +
 theme(plot.title = element_text(hjust = 0.5, size=10)) +
 labs(x="", y="")

 }
 else if (s > 515){
 p <- ggplot(subj_data, aes(x=SUBJ_OBS, y=SUBJ_PREDS)) + geom_point() +
 geom_abline(linetype = "dashed", color="blue") +
 geom_text(label= paste("R2 = ", round(m4_resamples$Rsquared[which(m4_resamples$Resample == paste("Resample", substr(as.character(s-1), 2,3), sep=""))],2), sep=""), x=0.25, y=3.75, size=2) +
 xlim(c(0,4.0)) +
 ylim(c(0,4.0)) +
 ggtitle(as.character(s)) +
 theme(plot.title = element_text(hjust = 0.5, size=10)) +
 labs(x="", y="")

 }

 plot_list[[i]] <- p
 i <- i + 1
 s <- s + 1
 }

(pg_1D <- plot_grid(plotlist=plot_list[1:9], ncol=3))
(pg_2D <- plot_grid(plotlist=plot_list[10:18], ncol=3))
(pg_3D <- plot_grid(plotlist=plot_list[19:27], ncol=3))
(pg_4D <- plot_grid(plotlist=plot_list[28:36], ncol=3))
(pg_5D <- plot_grid(plotlist=plot_list[37:45], ncol=3))
(pg_6D <- plot_grid(plotlist=plot_list[46:54], ncol=3))

***MODEL 5: OPENNESS TO EXPERIENCE***

STEP 1: BUILD AND RUN MODEL

library(caret)
library(xgboost)
library(boot)

#NUMBER OF SUBJECTS (N=54)
N <- length(unique(data_std$Ego_id))

#INITIATE LIST OBJECTS TO HOLD FOLD-BASED ROW INDICES BELONGING TO PERTINENT SUBJECTS
#MOST SUBJECTS ARE REPRESENTED 6 TIMES IN THE DATASET BECAUSE THERE ARE RMSSD OUTCOME VALUES FOR 6 SEPARATE WEEKS
list_of_training_idxs <- list()
list_of_validation_idxs <- list()

i <- 1
for (eid in unique(data_std$Ego_id)) {
 training_idxs <- which(data_std$Ego_id != eid)
 list_of_training_idxs[[i]] <- training_idxs

 validation_indices <- which(data_std$Ego_id == eid)
 list_of_validation_idxs[[i]] <- validation_indices
 i <- i + 1
 }

#REMOVE EGO_ID AND UNUSED OUTCOME FEATURES
test_data <- data_std[,-c(1,29,30,31,32)]

#RESET ROWNAMES TO AVOID SECRET SHENANIGANS
rownames(test_data) <- NULL

#SET TRAIN CONTROL VARIABLE AND SPECIFY ROW INDICES OF TRAINING DATA TO USE
set.seed(10111952)
seeds_5 <- vector(mode="list", length=55)
for(i in 1:54) seeds_5[[i]] <- sample.int(10000,36)
seeds_5[[55]] <- sample.int(10000,1)

fitControl_5 <- trainControl(method = "cv", #Note CV use set to N-1 folds; equivalent to LOOCV
 number = N-1, #Set folds
 savePredictions = TRUE,
 index = list_of_training_idxs,
 seeds = seeds_5)

#RUN TREE-BASED BOOSTING ENSEMBLE MODEL AND OPTIMIZE FOR R-SQUARED (% VARIANCE EXPLAINED)
model_out_5 <- train(CREA_RMSSD ~ ., data = test_data,
 method = 'xgbTree',
 metric = "Rsquared",
 trControl = fitControl_5)

#SAVE BEST-TUNED MODEL RESULTS
model_out_5_results <- data.frame(model_out_5$results[rownames(model_out_5$bestTune),])

#EXTRACT PREDICTIONS OF BEST-TUNED MODEL
m5_fold_predictions <- matrix(nrow=dim(data_in)[1], ncol=2)

m5_preds <- model_out_5$pred[which(model_out_5$pred$nrounds == model_out_5$bestTune$nrounds &
 model_out_5$pred$max_depth == model_out_5$bestTune$max_depth &
 model_out_5$pred$gamma == model_out_5$bestTune$gamma &
 model_out_5$pred$min_child_weight == model_out_5$bestTune$min_child_weight &
 model_out_5$pred$eta == model_out_5$bestTune$eta &
 model_out_5$pred$subsample == model_out_5$bestTune$subsample &
 model_out_5$pred$colsample_bytree == model_out_5$bestTune$colsample_bytree),]

#CREATE DATAFRAME TO STORE MODEL PREDICTIONS (COL 1) AND OBSERVED OUTCOME VALUES (COL 2)
for (row in (1:dim(data_in)[1])) {
 row_data <- m5_preds[which(m5_preds$rowIndex==row),]
 m5_fold_predictions[row,1] <- row_data$pred
 m5_fold_predictions[row,2] <- row_data$obs
}

model_out_5_results

STEP 2: SAVE IN-FOLD (SUBJECT-LEVEL) MODEL 5 PERFORMANCE

m5_resamples <- model_out_5$resample
m5_resamples_filtered <- m5_resamples[which(m5_resamples$Rsquared != 1.0),] #remove subjects with only two data points

par(mar = c(5, 5, 3, 5))
hist(m5_resamples_filtered$Rsquared, prob=FALSE, col="grey", breaks = 10, ylab="Count", xlab="R-squared", main="")

par(new=TRUE)
plot(density(m5_resamples_filtered$Rsquared), col="blue", lwd=2, xaxt="n", yaxt="n", main="", xlab="", ylab="", xlim=c(0,1))
axis(side=4)
mtext("Density", side=4, line=3)

STEP 3: CALCULATE MODEL 5 VARIABLE IMPORTANCE

(imp_5 <- varImp(model_out_5, scale=TRUE))

STEP 4: CREATE COWPLOTS TO ILLUSTRATE VARIABILITY IN PERFORMANCE ACROSS INDIVIDUALS

library(cowplot)

s <- 501
i <- 1
plot_list <- c()
for (val_list in 1:length(list_of_validation_idxs)) {
 subj_preds <- m5_preds[which(m5_preds$rowIndex %in% list_of_validation_idxs[[val_list]]),]$pred
 subj_obs <- m5_preds[which(m5_preds$rowIndex %in% list_of_validation_idxs[[val_list]]),]$obs
 subj_data <- data.frame(cbind(SUBJ_OBS=subj_obs, SUBJ_PREDS=subj_preds))

 if (s < 515) {
 p <- ggplot(subj_data, aes(x=SUBJ_OBS, y=SUBJ_PREDS)) + geom_point() +
 geom_abline(linetype = "dashed", color="blue") +
 geom_text(label= paste("R2 = ", round(m5_resamples$Rsquared[which(m5_resamples$Resample == paste("Resample", substr(as.character(s), 2,3), sep=""))],2), sep=""), x=0.25, y=3.75, size=2) +
 xlim(c(0,4.0)) +
 ylim(c(0,4.0)) +
 ggtitle(as.character(s)) +
 theme(plot.title = element_text(hjust = 0.5, size=10)) +
 labs(x="", y="")
 }
 else if (s == 515){
 s <- 516
 p <- ggplot(subj_data, aes(x=SUBJ_OBS, y=SUBJ_PREDS)) + geom_point() +
 geom_abline(linetype = "dashed", color="blue") +
 geom_text(label= paste("R2 = ", round(m5_resamples$Rsquared[which(m5_resamples$Resample == paste("Resample", substr(as.character(s-1), 2,3), sep=""))],2),sep=""), x=0.25, y=3.75, size=2) +
 xlim(c(0,4.0)) +
 ylim(c(0,4.0)) +
 ggtitle(as.character(s)) +
 theme(plot.title = element_text(hjust = 0.5, size=10)) +
 labs(x="", y="")

 }
 else if (s > 515){
 p <- ggplot(subj_data, aes(x=SUBJ_OBS, y=SUBJ_PREDS)) + geom_point() +
 geom_abline(linetype = "dashed", color="blue") +
 geom_text(label= paste("R2 = ", round(m5_resamples$Rsquared[which(m5_resamples$Resample == paste("Resample", substr(as.character(s-1), 2,3), sep=""))],2), sep=""), x=0.25, y=3.75, size=2) +
 xlim(c(0,4.0)) +
 ylim(c(0,4.0)) +
 ggtitle(as.character(s)) +
 theme(plot.title = element_text(hjust = 0.5, size=10)) +
 labs(x="", y="")

 }

 plot_list[[i]] <- p
 i <- i + 1
 s <- s + 1
 }

(pg_1E <- plot_grid(plotlist=plot_list[1:9], ncol=3))
(pg_2E <- plot_grid(plotlist=plot_list[10:18], ncol=3))
(pg_3E <- plot_grid(plotlist=plot_list[19:27], ncol=3))
(pg_4E <- plot_grid(plotlist=plot_list[28:36], ncol=3))
(pg_5E <- plot_grid(plotlist=plot_list[37:45], ncol=3))
(pg_6E <- plot_grid(plotlist=plot_list[46:54], ncol=3))

***POST-PROCESSING***

STEP 1: COMBINE AND ORDER VARIABLE IMPORTANCE SCORES ACROSS MODELS

names_1 <- rownames(imp_1$importance)
values_1 <- as.data.frame(imp_1$importance)
df_1 <- cbind(Variable=names_1, Importance=values_1)
rownames(df_1) <- NULL
df_1 <- df_1[order(df_1$Variable),]

names_2 <- rownames(imp_2$importance)
values_2 <- as.data.frame(imp_2$importance)
df_2 <- cbind(Variable=names_2, Importance=values_2)
rownames(df_2) <- NULL
df_2 <- df_2[order(df_2$Variable),]

names_3 <- rownames(imp_3$importance)
values_3 <- as.data.frame(imp_3$importance)
df_3 <- cbind(Variable=names_3, Importance=values_3)
rownames(df_3) <- NULL
df_3 <- df_3[order(df_3$Variable),]

names_4 <- rownames(imp_4$importance)
values_4 <- as.data.frame(imp_4$importance)
df_4 <- cbind(Variable=names_4, Importance=values_4)
rownames(df_4) <- NULL
df_4 <- df_4[order(df_4$Variable),]

names_5 <- rownames(imp_5$importance)
values_5 <- as.data.frame(imp_5$importance)
df_5 <- cbind(Variable=names_5, Importance=values_5)
rownames(df_5) <- NULL
df_5 <- df_5[order(df_5$Variable),]

ALL_IMP <- cbind(df_1, AGREE=df_2$Overall, CONSC=df_3$Overall, STABL=df_4$Overall, OPENN=df_5$Overall)
ALL_IMP <- cbind(ALL_IMP, AV_IMP=rowMeans(ALL_IMP[-1]))

ALL_IMP[order(ALL_IMP$AV_IMP, decreasing=TRUE),]

STEP 2: CODE SNIPPET TO CALCULATE MIN, MAX, MEAN R2 AND RMSE FOR EACH SET OF 5 IDIOGRAPHIC MODELS (RESAMPLE XX = PARTICIPANT)

resample <- "Resample01" #Change re-sample number

#R2
mean(c(m1_resamples$Rsquared[which(m1_resamples$Resample==resample)], m2_resamples$Rsquared[which(m2_resamples$Resample==resample)], m3_resamples$Rsquared[which(m3_resamples$Resample==resample)], m4_resamples$Rsquared[which(m4_resamples$Resample==resample)], m5_resamples$Rsquared[which(m5_resamples$Resample==resample)]))

max(c(m1_resamples$Rsquared[which(m1_resamples$Resample==resample)], m2_resamples$Rsquared[which(m2_resamples$Resample==resample)], m3_resamples$Rsquared[which(m3_resamples$Resample==resample)], m4_resamples$Rsquared[which(m4_resamples$Resample==resample)], m5_resamples$Rsquared[which(m5_resamples$Resample==resample)]))

min(c(m1_resamples$Rsquared[which(m1_resamples$Resample==resample)], m2_resamples$Rsquared[which(m2_resamples$Resample==resample)], m3_resamples$Rsquared[which(m3_resamples$Resample==resample)], m4_resamples$Rsquared[which(m4_resamples$Resample==resample)], m5_resamples$Rsquared[which(m5_resamples$Resample==resample)]))

#RMSE
mean(c(m1_resamples$RMSE[which(m1_resamples$Resample==resample)], m2_resamples$RMSE[which(m2_resamples$Resample==resample)], m3_resamples$RMSE[which(m3_resamples$Resample==resample)], m4_resamples$RMSE[which(m4_resamples$Resample==resample)], m5_resamples$RMSE[which(m5_resamples$Resample==resample)]))

max(c(m1_resamples$RMSE[which(m1_resamples$Resample==resample)], m2_resamples$RMSE[which(m2_resamples$Resample==resample)], m3_resamples$RMSE[which(m3_resamples$Resample==resample)], m4_resamples$RMSE[which(m4_resamples$Resample==resample)], m5_resamples$RMSE[which(m5_resamples$Resample==resample)]))

min(c(m1_resamples$RMSE[which(m1_resamples$Resample==resample)], m2_resamples$RMSE[which(m2_resamples$Resample==resample)], m3_resamples$RMSE[which(m3_resamples$Resample==resample)], m4_resamples$RMSE[which(m4_resamples$Resample==resample)], m5_resamples$RMSE[which(m5_resamples$Resample==resample)]))
