# Supplementary material for: Using passive sensor data to probe associations of social structure with changes in personality: A synthesis of network analysis and machine learning
Source: PLoS One. 2022 Nov 30;17(11):e0277516. doi: 10.1371/journal.pone.0277516 (PMC9710841; doi:10.1371/journal.pone.0277516)
Supplement: S5 File — (DOCX) [file pone.0277516.s005.docx]

**Table S1. Comparative Performance of Disparate Machine Learning Algorithms**


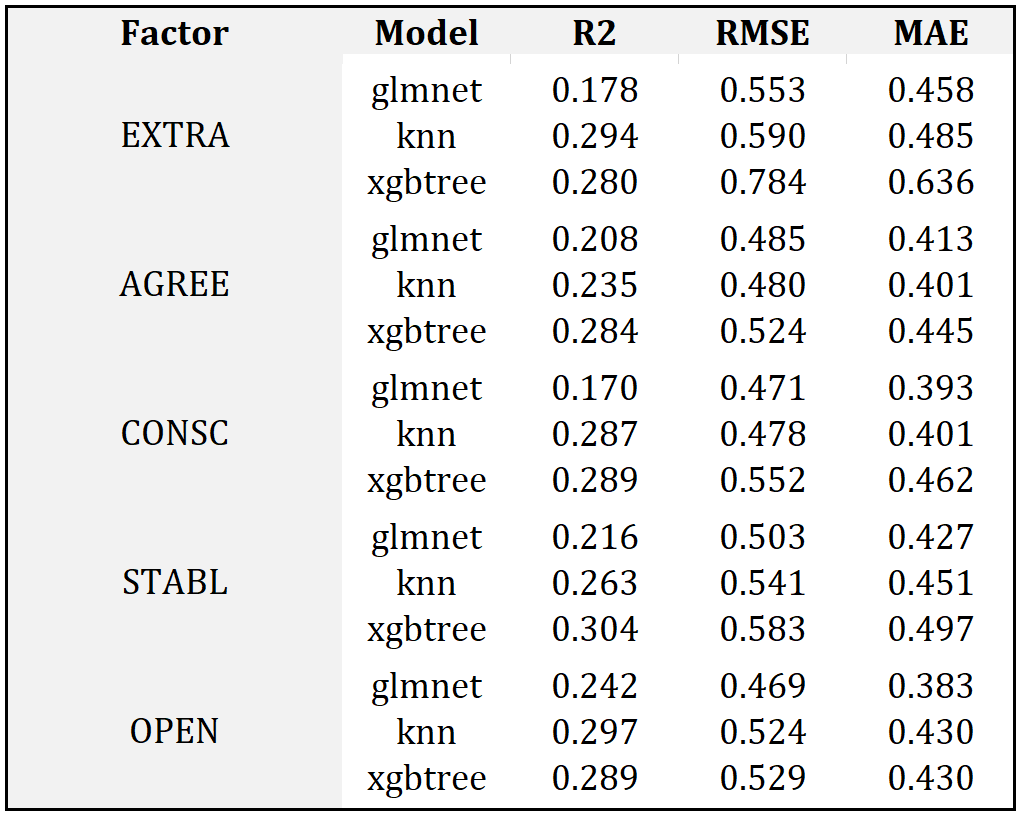


EXTRA = extraversion; AGREE = agreeableness; CONSC = conscientiousness; STABL = stability; OPEN = openness to experience; glmnet = generalized linear model with elastic net penalty; knn = *k­*-nearest neighbors; R2 = variance explained; RMSE = root mean square error; MAE = mean absolute error.
