# Supplementary material for: Using passive sensor data to probe associations of social structure with changes in personality: A synthesis of network analysis and machine learning
Source: PLoS One. 2022 Nov 30;17(11):e0277516. doi: 10.1371/journal.pone.0277516 (PMC9710841; doi:10.1371/journal.pone.0277516)
Supplement: Attachment — Submitted filename: plos_review1.docx [file pone.0277516.s006.docx]

This work leverages data from a previously published study to combine passively collected wearable sensor information on face-to-face, workplace social interaction with an ecological momentary assessment of personality state. Data from 54 individuals across six weeks was used to explore the relative importance of social network structural features in predicting changes in the Big Five (5F) personality states. Using only SNA-derived features from wearable sensor data, models explained, on average, approximately 28-30% of the variance in an individual personality state change. Model introspection implicated egocentric features as the most influential predictors across 5F-specific models with network efficiency, constraint, and effective size measures among the most important. Feature importance profiles for each 5F model partially echoed previous empirical findings. Results support future efforts focusing on egocentric components of SNA and suggest particular investment in exploring efficiency measures to model personality fluctuations within the workplace setting.

My comments are as follows:

1. The abstract can be re-written to better explain the methodology applied/used/developed in the paper.

2. A better way to describe subsection 2.2 "Data preprocessing and outcome operationalization" is to include a table and list out the parameters and their ranges. Also, discuss the performance-metrics description (mathematical formulas) used to evaluate the proposed approach.

3. There are so many sections with little details. Combine them appropriately and include only the relevant ones.

4. The result section needs significant improvement in terms of better discussion regarding the performance analysis of the proposed approach.

5. The quality of the figures requires improvement. At present many of them are quite blurry.
